# Living on the sea-coast: ranging and habitat distribution of Asiatic lions

**DOI:** 10.1038/s41598-022-23761-1

**Published:** 2022-11-10

**Authors:** Mohan Ram, Aradhana Sahu, Shyamal Tikadar, Harshal Jayawant, Lahar Jhala, Yashpal Zala, Meena Venkataraman

**Affiliations:** 1 Wildlife Division, Sasan-Gir, Junagadh, Gujarat 362 135 India; 2 Wildlife Circle, Junagadh, Gujarat 362 001 India; 3 Office of the Chief Wildlife Warden, Gujarat Forest Department, Gandhinagar, Gujarat 382 010 India; 4 Carnivore Conservation & Research (CCR), Mumbai, Maharashtra India

**Keywords:** Conservation biology, Grassland ecology, Population dynamics

## Abstract

Endangered Asiatic lions (*Panthera leo persica*) are renowned for their resilience and as a flagship of successful conservation and management. Lions dispersing out of the Gir forest have established themselves in the coastal habitats for about 25 years. We propose that the home range and spatial distribution of lions inhabiting the coastal habitats would be distinct from the forested habitats of the protected area. Each individual was monitored for an average of 367.2 ± 99.05 days from 2019 to 2021. The mean core area was 33.8 km^2^ (50% FK, SE 8.7 km^2^) and the overall average range was 171.8 km^2^ (90% FK, SE 40.5 km^2^). The home ranges were significantly larger for lions residing in the coastal area compared to lions in the protected area. The lion distribution model was built on MaxEnt, and inputs included location fixes of lions and variables, including 18 land use categories and Euclidean distance to linear infrastructures and human settlements. Lions were shown to use forest habitat patches extensively, followed by available habitats around water sources and wasteland. The study highlights the importance of corridors connecting to the Gir protected area and the importance of coastal forest patches for lion conservation and management.

## Introduction

Large carnivores are territorial and require large spaces and a good prey base to survive^1,2^. A good understanding of carnivore ecology is fundamental for conservation planning to meet their requirement for large spaces and a good prey base^1–4^. When carnivores, particularly lions, disperse out of designated protected areas, it becomes crucial to reassess their ranging and habitat requirements specific to these heterogeneous land uses in order to implement appropriate conservation and management strategies.

**Figure 1:**
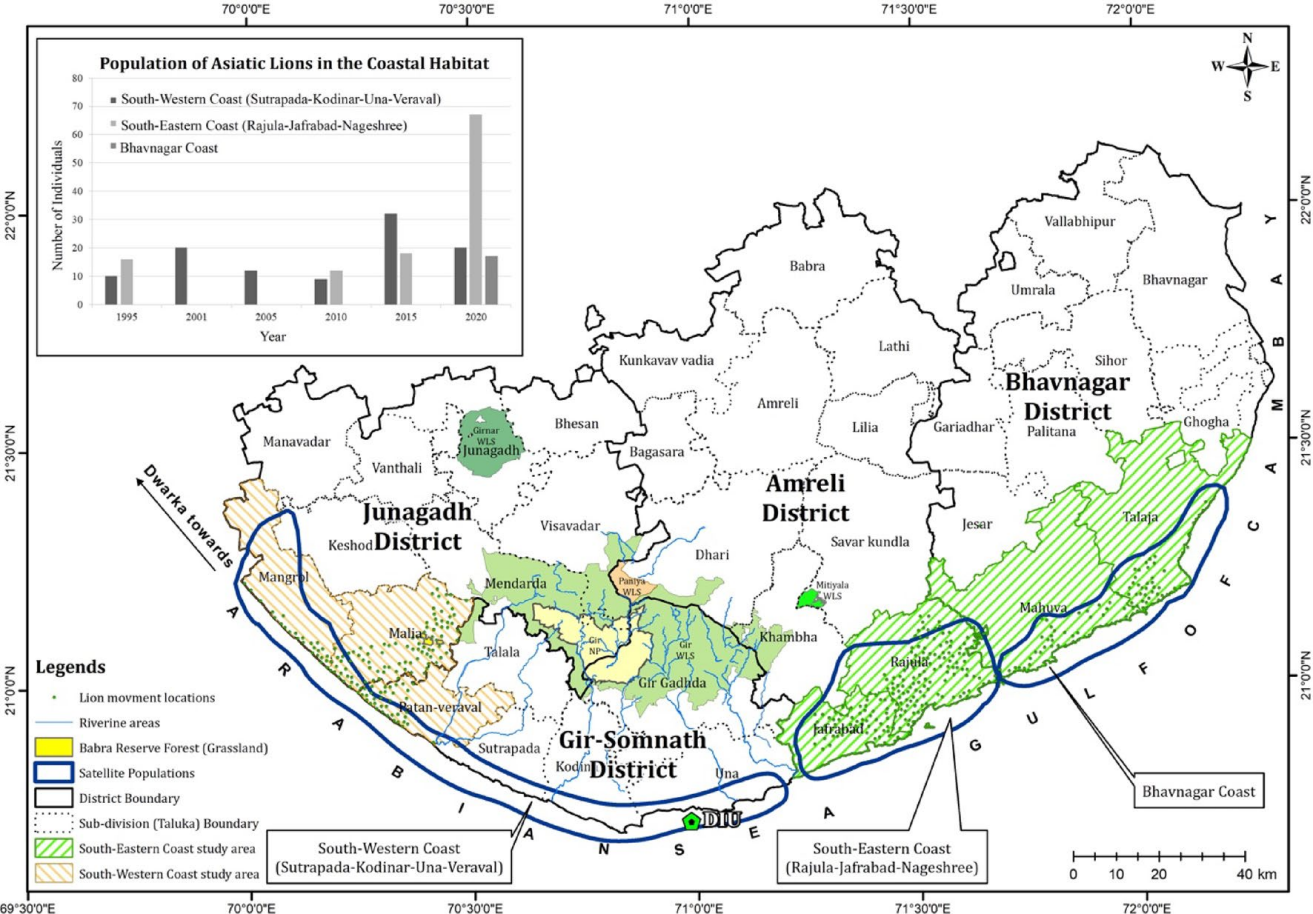
Location of the eastern and western coastal habitats in the Asiatic Lion Landscape in Gujarat. The inset shows the population of lions in the coastal habitats.

Across their range, lions are known to occupy a wide range of habitat types^5,6^. Within their current range, the endangered Asiatic lions utilize landscapes of varied terrain, vegetation, and land use^6,7^. This situation is an outcome of around a four-fold increase in lion population since 1968, and around 48% of the present population of 674 lions (June 2020) have dispersed out of the protected areas, ranging across nine districts and 13 forest administrative divisions^8,9^. Lions occupying natural habitats outside the Gir Protected Area (Gir PA) have been described as satellite populations residing in varying satellite habitats, including undulating terrains, riverine areas and coastal habitats^10^. The most significant of the satellite habitats occupied by dispersed lions are coastal habitats. The first record of lions in the coastal habitats of Sutrapada was in the mid-1990s, and since then, there has been a continuous presence of lions in the coastal areas that extend across four districts (Fig. 1)^7,10^.

We propose that the home range and spatial distribution of lions inhabiting the coastal habitats would be distinct from the forested habitats of the protected area. We studied the spatial ecology of 10 lions from 2019 to 2021 by equipping them with GPS radio-collars. Our study had the following components, (i) Estimation of home range and core areas of lions residing in the coastal habitats, (ii) Comparison of the home range of lions inhabiting forested habitats (Gir PA) and coastal habitats and also with transient lions moving between the two habitats (link lions), (iii) lion distribution model to understand how lions are surviving in the coastal habitats.

## Results

The 10 lions, comprising 3 adult males, 4 adult females, 2 sub-adult females, and 1 sub-adult male, were monitored from 2019 to 2021 for an average of 367.2 (SD 99.05, range 185–467) days (Table 1). Three malfunctioned radio-collars were replaced, and all the radio-collars were removed at the end of the monitoring period by using an inbuilt drop-off function.

**Table 1:** Age-sex details of radio-collared lions, number of days monitored, home range (90% Fixed Kernel estimate) and core area (50% Fixed Kernel estimate). Lions ranging exclusively in the coastal habitats, in the landscape between Gir PA and coast, exclusively within Gir PA are categorized as ‘Coastal’, ‘link’ and ‘protected area’ respectively to compare their home range sizes.

Sr. No	Sex	Monitor days	Overall home range (km^2^)	Core area (km^2^)	Ranging category
1	AM	399	323.36	63.76	Coastal
2	SAF	456	90.07	8.43	Coastal
3	AM	397	189.58	29.34	Coastal
4	AF	254	273.30	74.74	Coastal
5	AM	467	68.64	16.81	Coastal
6	AF	185	2023	4.12	Coastal
7	AF	332	319.52	67.88	Coastal
8	SAM	451	325.81	49.94	Coastal
9	SAF	450	44.03	11.32	Coastal
10	AF	281	63.93	11.99	Coastal
11	SAM	451	719.00	192.90	Link
12	SAM	382	923.64	220.75	Link
13	SAM	452	325.81	49.94	Link
14	AF	199	62.27	17.16	PA
15	AF	457	35.67	8.18	PA
16	AM	111	60.17	21.06	PA
17	AM	136	9.53	5.19	PA
18	AM	31	13.00	3.62	PA
19	AM	249	75.57	10.80	PA
20	AF	457	151.45	26.41	PA
21	AM	340	117.54	22.09	PA

*AM* Adult male, *AF* adult female, *SAM* sub-adult male, *SAF* sub-adult female, *PA* protected area.

### Home range

**Figure 2:**
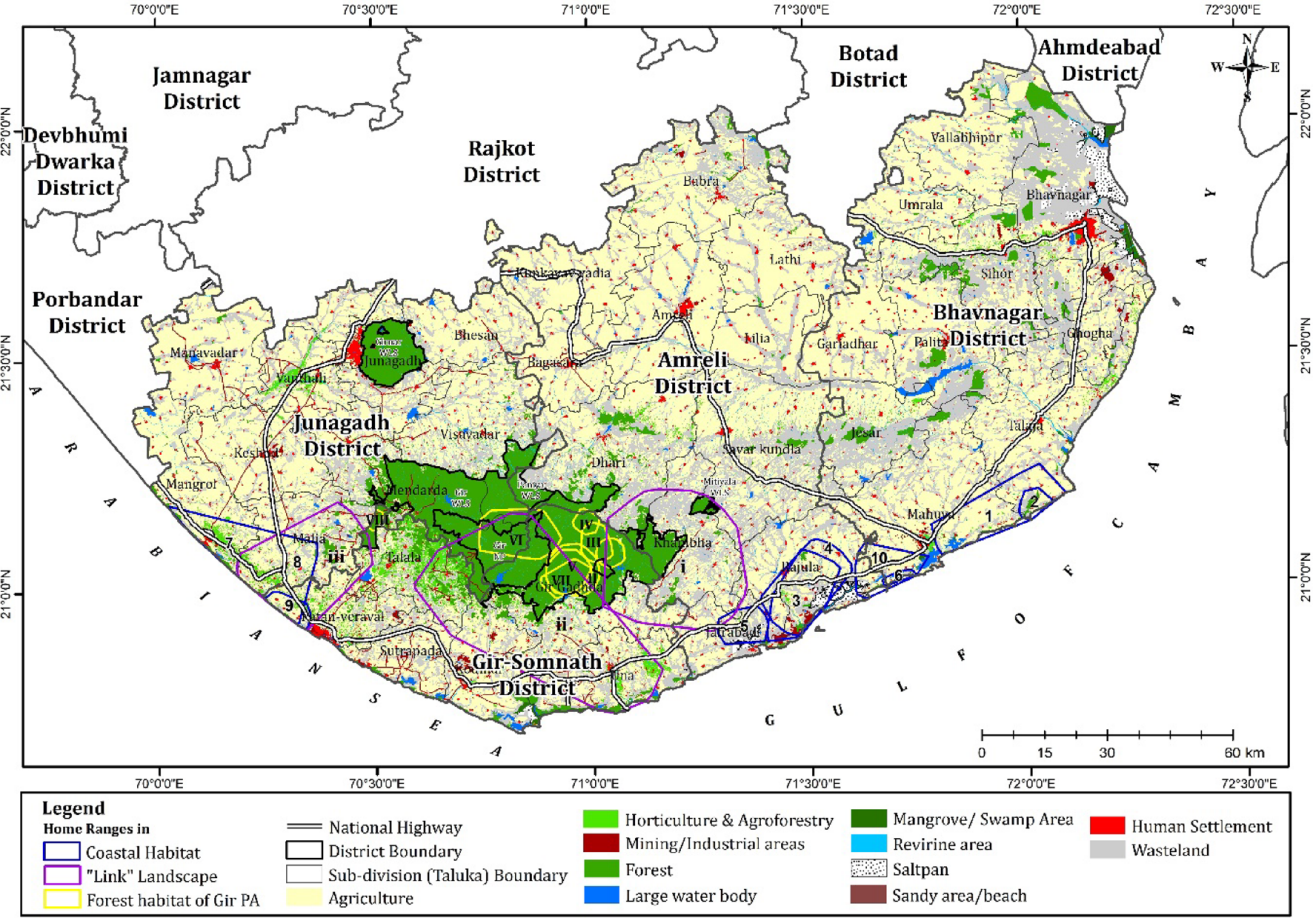
Land Use Land Cover (LULC) of eastern and western coastal lion habitats. The figure also indicates the distribution of radio-collared lions ranging in the (i) coastal habitats, (ii) “link” landscape between the Gir PA and the coastal habitats, and (iii) forested habitats of the Gir PA.

The overall mean home range size (90% FK) of the coastal lions (N = 10) was 171.8 km^2^ (SE 40.5 km^2^), while the mean core area (50% FK) was 33.8 km^2^ (SE 8.7 km^2^). Individual home ranges varied from 44.3 to 325.8 km^2^, while the core areas ranged from 8.4 to 67.8 km^2^ (Table 1, Fig. 2). The mean home range size of adult female (N = 4) was 214.76 km^2^ (SE 55.7 km^2^), adult male (N = 3) was 193.86 km^2^ (SE 73.5 km^2^), sub-adult female (N = 2) was 67.05 km^2^ (SE 23.02 km^2^) and sub-adult male (N = 1) was 325.8 km^2^.

The lions (N = 3) ranging in the “link landscape” (the landscape between Gir PA and coastal habitats) had a mean home range size of 656.2 ± 303.8 km^2^ and a core area of 154.5 ± 91.6 km^2^ (Fig. 2).

The lions ranging in the forested Gir PA (N = 8) had a mean home range of 65.6 ± 49.3 km^2^ and a core area of 14.3 ± 8.5 km^2^ (Fig. 2).

The home ranges of lions in the coastal regions are greater than the home ranges of lions within Gir PA, while the home ranges of lions ranging between Gir PA and coastal habitat (“link lions”) were greater than the home ranges of the coastal lions. The Lion home range in forested habitats of Gir PAs was significantly different from coastal habitats (p = 0.03, Z = 2.21, U = 59). There was no significant difference in lions’ core ranges in forest and coastal habitats.

### Habitat use and lion distribution

**Figure 3:**
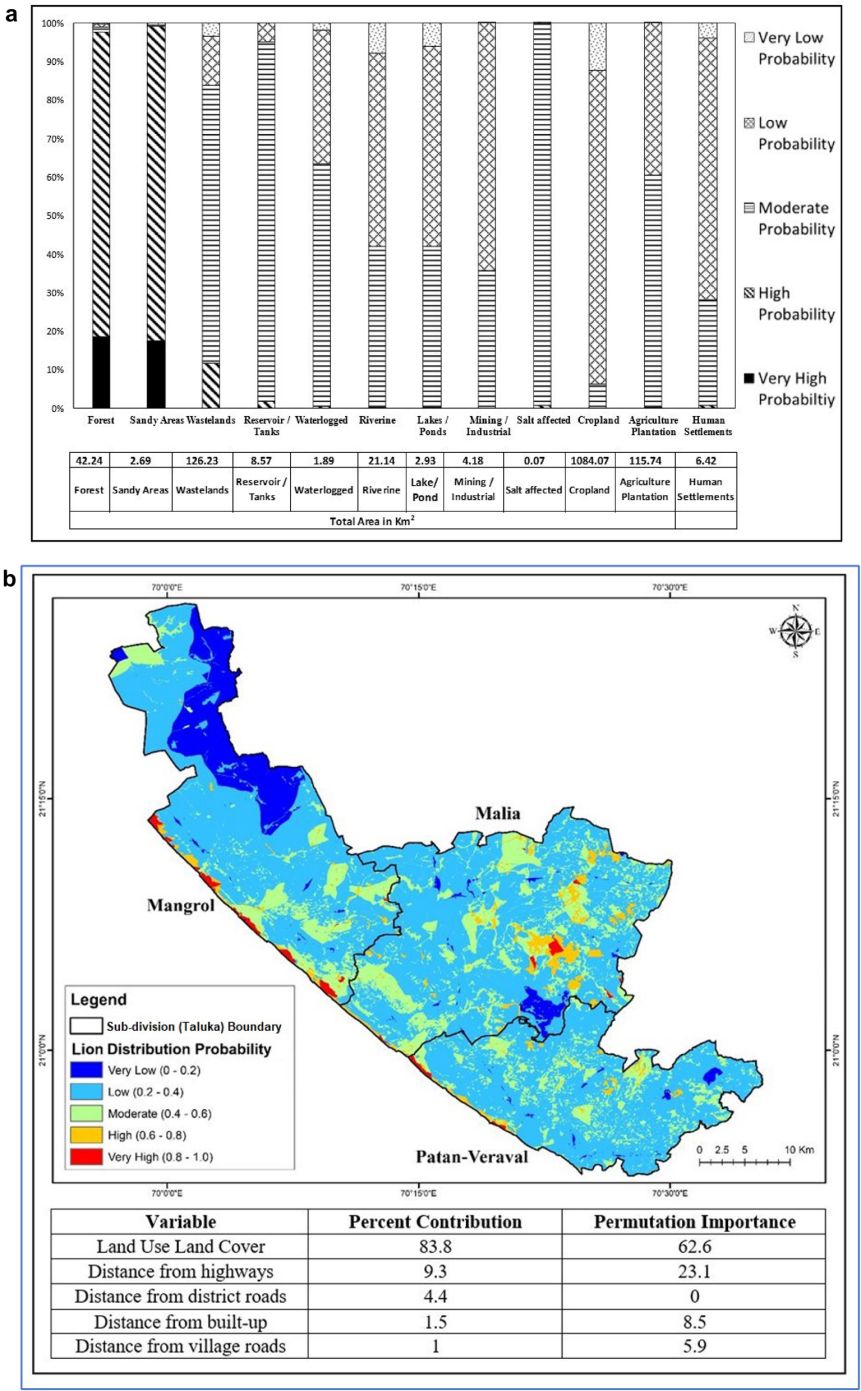
(**a**) The proportion (%) of very low to high probability areas under each LULC category in the western coastal lion habitat. The table indicates the total area (km^2^) under each category in the western coastal lion habitat of the study area, covering 1413 km^2^. (**b**) Lion distribution map of lions ranging in the western coastal habitat based on MaxEnt models. Red colours indicate a higher “probability of occurrence” (suitability), while blue colours indicate lower probabilities. Using the MaxEnt logistic output, the percent contribution and permutation Importance are shown in the table. LULC was found to contribute the majority (83.8%).

**Figure 4:**
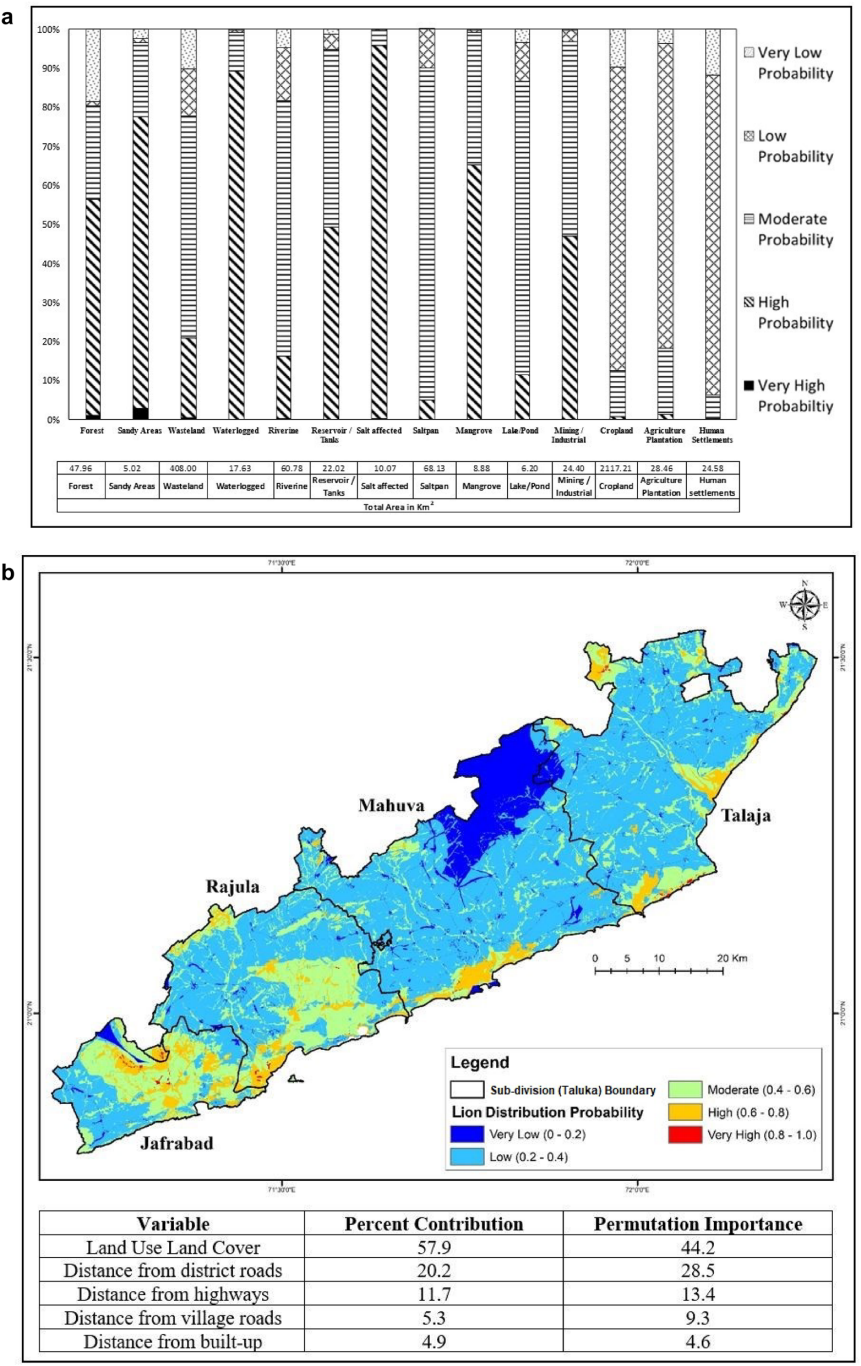
(**a**) The proportion (%) of very low to high probability areas under each LULC category in the eastern coastal lion habitat. The table indicates the total area (km^2^) under each category in the eastern coastal lion habitat of the study area, covering 1413 km^2^. (**b**) Lion distribution map of lions ranging in the eastern coastal habitat based on MaxEnt models. Red colours indicate a higher “probability of occurrence” (suitability), while blue colours indicate lower probabilities. Using the MaxEnt logistic output, the percent contribution and permutation Importance are shown in the table. LULC was found to contribute the majority (57.9%).

Model efficiency for the west coast lion distribution model in terms of the training data AUC value was 0.73, and the test data AUC value was 0.65. For the east coast, the training data AUC value was 0.72, and the test data AUC value was 0.69. Land use land cover (LULC) was the most significant contributor for both the western (83.8%) and eastern (57.9%) coast, followed by other variables (Figs. 3b, 4b).

### Western coast

Natural land cover, namely forests (0.79), sandy areas (0.77) and open scrubland (0.59), showed a high probability of lion distribution taking other variables of the model at their average sample value (Table 2). Village roads, district roads and highways showed a low probability of occurrence. The effect of roads was examined at a 3 km distance, and lion distribution probability was found to increase with distance in the case of highways (Supplementary Fig. 1A,B). Croplands (0.37), agriculture plantations (0.49) and various categories of human settlement such as peri-urban, urban and villages (0.49) showed a low probability of lion distribution (Table 2).

**Table 2:** Lion distribution probability (logical output of MaxEnt model) for each Land Use Land Class (LULC) type for the eastern and western coast. Response A is the lion distribution probability for the LULC variable while keeping other variables at average value, while Response B is the distribution probability considering only the LULC variable.

Sr. no.	LULC type	Lion distribution probability			
		East coast		West coast	
		Response A	Response B	Response A	Response B
1	Forest	0.68	0.70	0.79	0.78
2	Sandy areas	0.69	0.68	0.77	0.79
3	Dense scrubland	0.72	0.75	0.49	0.43
4	Open scrubland	0.56	0.56	0.59	0.54
5	Salt affected	0.72	0.73	0.00	0.00
6	Saltpan	0.56	0.52	Not present	Not present
7	Mangrove	0.65	0.67	Not present	Not present
8	Lakes/ponds	0.56	0.53	0.49	0.43
9	Riverine	0.58	0.61	0.47	0.43
10	Waterlogged	0.65	0.71	0.00	0.00
11	Reservoir	0.56	0.53	0.49	0.44
12	Mining	0.59	0.59	0.49	0.43
13	Cropland	0.36	0.36	0.37	0.32
14	Agriculture plantation	0.37	0.36	0.49	0.43
15	Core Urban	0.56	0.53	0.49	0.43
16	Village	0.56	0.37	0.49	0.39
17	Mixed settlement	0.56	0.53	0.49	0.43
18	Peri urban	0.00	0.00	0.49	0.43

Forests and sandy areas had a high percentage of both high and very high probability areas of lion distribution. In contrast, croplands and human settlement areas had a high proportion of low probability areas (Fig. 3a,b).

### Eastern coast

Natural land cover, namely forests (0.68), dense scrubland (0.72), salt-affected (0.72) and sandy areas (0.69), showed a high probability of lion presence (Table 2). The effect of roads was examined at a 3 km distance, and lion distribution was found to increase with distance. Village roads, district roads and highways showed a low probability of occurrence (Supplementary Fig. 2A,B). Croplands (0.36), agriculture plantations (0.37) and various categories of human settlement such as core-urban (0.56) and villages (0.56) (Table 2) showed a low probability of lion distribution.

Forests and sandy areas had a high percentage of high and very high probability areas of lion distribution. In contrast, croplands and human settlement areas had a high proportion of low probability areas (Fig. 4a,b).

## Discussion

Mammalian carnivores have suffered significant range contraction and sharp population declines worldwide^4,11^. Across their range, lions in Africa do well in some areas, but in others, their status is precarious^5^. Whereas the population of the Asiatic lions has shown steady growth in the past six decades owing to dedicated conservation management and support of the local people^8,12^. These efforts have led to the down-listing of the Asiatic lion’s status from ‘Critically Endangered’^13^ to ‘Endangered’ category by the IUCN red-listing^14^. Another outcome of this population growth is the dispersal of lions outside Gir PA in the Asiatic Lion Landscape^8,9^.

There are 20 lions on the southwestern coast, 67 lions on the south-eastern coast and 17 lions on the Bhavnagar coast^8^. These demographics and data from previous population estimates confirm that the coastal populations are a vital and growing metapopulation (Fig. 1). This is the first systematic study on lions inhabiting the coastal habitats. In this study, 15% of the adult coastal lion population has been intensively monitored to understand their ranging and habitat use.

The study indicates that the home ranges in coastal habitats are significantly larger than lion home ranges within Gir PA. This could be due to dispersed resources in the patchily distributed preferred refuge habitats (Fig. 2). It is well known that lion ranging is primarily influenced by habitat, water availability, prey distribution and availability^15,16^. The ecological drivers for this observed difference in home ranges habitats is a matter of further research.

Lion home ranges during the nomadic phase of their social life are also known to be large and unpredictable in addition to their movement in relation to resource needs^16–18^. Three lions designated as “link” individuals had a significantly larger range and were seen to be using some important forest patches in the intermediate space as stepping stones. The patches include reserved grasslands (locally known as *vidis*) such as Babara *vidi*^19^ (Fig. 1). There are 17 riverine corridors originating from the western boundary of Gir PA, ending at the sea coast^12^. The grassland patches have high habitat suitability and are identified as high lion distribution probability areas by the MaxEnt model (Figs. 3a,b, 4a,b). However, the available coastal habitat patches for lions that cover an area of 110 km^2^ are scattered across 415 km^2^ among 33 villages in three districts (excluding the newly occupied Bhavnagar coast)^7^. The present study provides data to specifically map these stepping zone habitats and corridors that are now identified as being vital for maintaining viable sink populations for similar scenarios^20^. The link lions are proof that there is movement between the protected area and coastal lion habitats.

The forest land classes show a high probability of lion distribution in the western (probability 0.79) and eastern (0.68) coastal habitats (Table 2). In all, 70 km^2^ fall under other forest categories, including Reserved forests and Unclassed forests encompassing 23 villages, forming the coastal border spreading across Kodinar, Una, Rajula and Jafrabad^21^. The sandy-shore areas with high lion distribution probability of 0.77 and 0.69 for western coastal and eastern coastal habitats, respectively, are important, particularly for the movement of lions between forest patches (Figs. 3a,b, 4a,b). Apart from these, the wastelands LULC, which denotes both open and dense scrublands, are important as stepping stones and part of the home range and core areas for lions of the coastal habitats. The forest patches, sandy coastal areas, and wastelands are important components of the lion home range (Fig. 2, Table 2).

While the western coastal areas have a long stretch of *Prosopis juliflora* along the coast, showing very high probability areas (0.8–1.0), the eastern coast has more of the typical coastal vegetation, namely mangroves, salt pans and salt-affected areas that occur in patches. This may be the reason for the relative differences in the distribution probability (Figs. 3b, 4b). This study shows that 60% of the available 8 km^2^ mangroves and swamps areas have a high probability of lion distribution (Table 2, Figs. 2, 4a,b). Salt affected (0.74 probability), salt pan (0.56) and mangroves (0.65) cover about 87 km^2^ of the study area and are important for the lions ranging in the eastern coastal areas (Table 2, Figs. 2, 4a,b). This emphasizes the importance of these areas for the resident breeding population of lions.

The cropland, agriculture plantation and human settlement areas consistently have a low probability of lion distribution (Table 2, Figs. 3a, 4a). Understandably, the presence of a high density of road networks, including village roads, district roads, and State and National highways, is an important factor in the lion probability distribution model. This is because lions tend to use these land use features, particularly the village and district roads, as movement corridors. Therefore, linear infrastructure projects should be carefully planned with proactive mitigation measures based on this understanding. Human land use areas are indicated as having a low probability of lion distribution areas. However, they occupy a substantial part of the lion home ranges (Fig. 2), emphasizing that all aspects of development activity and human-lion interactions should be incorporated into management planning^7^.

The survival of lions on the sea coast is possible because both the stepping stone patches and corridors for dispersing lions, as well as the lion home ranges, have natural vegetation patches—a large proportion of which are under various categories of legally protected areas. These patches and the typical coastal habitats, such as the saltpans and sandy shore areas, are important components of lion home ranges and should be protected (Table 1).

## Conclusion

Our study helps managers to evaluate the landscape-level management strategies for conserving the Asiatic lions by (1) identifying linkage habitats for corridor connectivity and gene flow by modelling the spatial distribution of lions. Going forward, through the protection of these highlighted natural habitat patches along the coast and those linking with the protected area should be monitored and protected, (2) The significance of LULC of the landscape by monitoring ranging and space-use by lions occupying the coastal habitats. We indicate the importance of maintaining the integrity of this LULC. We demonstrate how a study of spatial ecology is critical to demarcate movement corridors and habitat requirements of lions in the existing land-use matrices for a meta-population or landscape approach to conservation^22^.

## Materials and methods

### Study area

Situated in western India’s southwestern part of the Gujarat state, the Saurashtra region typically represents the semi-arid Gujarat-Rajputana province 4B^23^, which covers 11 out of 33 districts of the state. The region forms a rocky tableland (altitude 300–600 m) fringed by coastal plains with an undulating central plain broken by hills and dissected by various rivers that flow in all directions^24^. With the longest coastline (~ 1600 km) in India, Gujarat is endowed with rich coastal biodiversity^25,26^. The Saurashtra coast in Gujarat is encircled by the open sea between two Gulfs (68° 58′–71° 30′ N and 22° 15′–20° 50′ E) and divided into two segments, viz*.* the southwestern coast from Dwarka to Diu (~ 300 km stretch) and south-eastern coast from Diu to Bhavnagar (~ 250 km stretch)^26^.

The Asiatic Lion Landscape covers an area of ~ 30,000 km^2^ (permanent lion distribution range: ~ 16,000 km^2^; visitation record range: ~ 14,000 km^2^) of varied habitat types within Saurashtra. The landscape includes five protected areas (Gir National Park, Gir Wildlife Sanctuary, Paniya Wildlife Sanctuary, Mitiyala Wildlife Sanctuary, and Girnar Wildlife Sanctuary) and other forest classes (reserved forests, protected forests, and unclassed forests).

The coastal habitats extend across the districts of Bhavnagar, Amreli, Gir-Somnath, and Junagadh (Fig. 1). Within these districts (Fig. 1), the tehsils (sub-divisions/taluka) of Mangrol, Malia, Patan-Veraval, Sutrapada, Kodinar and Una are categorized under the southwestern coast (hereafter western coastal habitat), Jafrabad, Rajula, form the south-eastern coast and Mahuva and Talaja constitute the Bhavnagar coast and represent distinct lion range units (Fig. 1). The total area covered in the study is 2843 km^2^ on the eastern coast and 1413 km^2^ on the western coast (Fig. 1).

The Saurashtra region is bestowed with three distinct seasons, viz*.* dry and hot summer (March–June), monsoon (July–October), and primarily dry winter (November–February). It receives a mean annual rainfall of ~ 600 mm, with most rainfall during the southwest monsoon^27^. The mean maximum and minimum temperatures are 34 °C and 19 °C, respectively^28^. There is a 110 km^2^ stretch of forests along the coast. The rest of the areas are multi-use consisting of private, industrial, pastoral and wastelands of varied ownerships. The natural vegetation primarily consists of *Prosopis juliflora* and *Casuarina equistsetifolia*. On the beach and dune areas, vegetation such as *Ipomea pescaprae*, *Sporobolus trinules*, *Fimrystylis* sp., *Crotalaria sp.*, and *Euphorbia nivuleria*^29^. The mudflats along the coast are restricted to Talaja, Mahuva, Pipavav Port, Jafrabad creek, and Porbandar, sparsely covered by the *Avicennia marina*^29^. Fisheries, agriculture, horticulture, livestock rearing, and some large- and small-scale industries are the leading economies in the coastal belt.

Coastal segments are characterized by the variety of vegetation, sandy beaches, small cliffs, wave-cut platforms, open and submerged dunes, minor estuaries, embankments, and transition from the open sea to gulf environment with tidal mud^26,29^ and also support a diverse assemblage of biodiversity^25^. This biodiversity is further enriched by several perennial/ephemeral rivers originating from the Gir PA (Shetrunji, Machundari, Raval, Ardak, Bhuvatirth, Shinghoda, Hiran, Saraswati, etc.)^12^. These rivers meet the sea at different sections of the coast, forming prominent coastal ecosystems^25^. The riverine tracts act as important corridors for wildlife movement^9,12,30^. Dispersing through these corridors, lions have started inhabiting these coastal habitats^30,31^.

### Methods

All the research activities involved in this study on Asiatic lions were carried out after taking due permission from the Ministry of Environment, Forests & Climate Change (MoEF&CC), Government of India (Letter No.: F. No. 1-50/2018 WL) and Principal Chief Conservator of Forests (Wildlife) & Chief Wildlife Warden, Gujarat State, Gandhinagar (Letter No.: WLP 26B 781-83/2019-20). Procedures and protocols were followed as per the Standard Operating Procedures of the Gujarat Forest Department, Government of Gujarat, concerning the handling of wild animals. Qualified and experienced veterinarians and their team carried out all procedures related to radio-collaring. Moreover, the study is reported in accordance with ‘Animal Research: Reporting of In Vivo Experiments’ (ARRIVE) guidelines as applicable.

A long-term lion monitoring project was initiated in 2019 by the Gujarat Forest Department to understand the movement patterns and ecology of lions in the Asiatic Lion Landscape. Looking at the heterogeneity and vastness of the coastal areas, ten individuals were carefully selected for satellite radio-collaring based on their frequent movement in different coastal habitats and monitored from 2019 to 2021.

The lions were deployed with Vertex Plus GPS Collars (Vectronics Aerospace GmbH, Berlin, Germany) that weighed less than three per cent of the individual’s body weight, irrespective of age and sex. The lions were immobilized using a combination of Ketamine hydrochloride (2.2 mg per kg body weight; Ketamine, Biowet, Pulawy) and Xylazine hydrochloride (1.1 mg per kg body weight; Xylaxil, Brilliant Bio Pharma Pvt. Ltd., Telangana)^32^ administered intramuscularly using a gas-powered Telinject™ G.U.T 50 (Telinject Inc., Dudenhofen, Germany) dart delivery system. A blindfold was placed to protect the eyes and decrease visual stimuli^33,34^. Each sedated individual was sexed, aged, and measured as per the standard operating procedure (SOP) of the Gujarat Forest Department, Government of Gujarat, and recorded the data in the trapping datasheet. The radio-collars were deployed considering the neck girth of the individual, ensuring free movement of it so as not to hamper the individual’s routine activities. After deploying the radio-collar, we used the specific antidote for Xylazine, i.e., Yohimbine hydrochloride (0.1–0.15 mg per kg body weight; Yohimbe, Equimed, USA) intravenously, resulting in the total recovery of immobilized individuals^32^ within 5–10 min. The individuals were intensively monitored for 72 h and, after that, regularly monitored throughout the functional period of the radio-collars. The entire radio-collaring exercise was carried out by trained and experienced veterinary officers and their teams that constituted wildlife health care personnel and field staff.

Each collar had a unique VHF and UHF frequency. The radio-collars were equipped with a programmable GPS schedule and configured to record the location fixes at every hour and provided the data through the constellation of low-earth-orbit Iridium satellite data service (Iridium Communications Inc., Virginia, USA) at four-hour intervals after getting activated. The data logs included location fixes in degree decimal format (latitude/longitude), speed (km/hour), altitude (meters above mean sea level), UTC timestamp (dd-mm-yyyy h:m:s), direction (degrees), and temperature (Celsius). Radio-collars were equipped with mortality sensors and a programmable drop-off activation system. Gir Hi-Tech Monitoring Unit, Sasan-Gir, Gujarat, monitored and coordinated these activities. The location data from each radio-collar was downloaded using the GPS Plus X software (Vectronics Aerospace GmbH, Berlin, Germany) in the Gir Hi-Tech Monitoring Unit (a technology-driven scientific monitoring initiative in the landscape established in 2019 at Sasan-Gir, Gujarat).

### Data analysis

In this study, we calculated the home range of lions resident in the coastal region using the Fixed Kernel method. We expressed them as 90% and 50% Fixed Kernel (FK) to summarize the overall home range and core area, respectively^35–37^. Additionally, the home range of lions categorized as “link lions” and lions of the protected area was summarized for comparison (Table 1).

MaxEnt (version 3.4.1) stand-alone software^38^ was applied for fine-scaled lion distribution modelling^39,40^. The logistic output format was set for the MaxEnt output. 30% random lion occurrence points were used as test data to evaluate model performance. The area under the receiver operating characteristic curve (AUC) was used to evaluate the discriminative ability of the model based on the values of sensitivity (correct discrimination of true positive location points) and specificity (correct discrimination of true negative absence points)^41^. The Jackknife regularised training gain for the species was used to understand the effect of each variable in model building. The logical output by the MaxEnt was presented in a table format as “percent contribution” and “permutation importance” values (from 0 to 100%). Spatial inputs were prepared on the GIS platform using ArcMap (version 10.8.1, ESRI, Redlands, USA)^42^. Input data for MaxEnt were categorized as (i) lion occurrence data, (ii) model variables were prepared as described below:

i. Occurrence data
  At the first level, inconsistent location fixes (records with missing coordinates, time stamps, and elevation) and outliers were filtered out. Next, each lion’s hourly GPS location fixes obtained from remotely monitored radio-telemetry data were randomized to overcome spatial and temporal biases. The data was reduced by taking every three-hour location fix^43,44^. The data was further categorized season-wise, viz*.* summer, monsoon and winter. This consolidated data was then subject to spatial thinning of one kilometre using SDMtoolbox (version 2.0)^45,46^.
ii. Model variables

The variables used for distribution modelling broadly included different categories of land use, including both natural habitats and anthropogenic factors, namely, roads and human settlement areas. All variables were rasterized at 10 m spatial resolution.

Land Use Land Cover (LULC) data of Saurashtra was obtained from Bhaskaracharya National Institute for Space Applications and Geo-informatics (BISAG-N), Gandhinagar, Gujarat. The data was then further classified into 18 sub-classes—Forest, Sandy areas, Salt-affected, Saltpan, open scrub, dense scrub (Wastelands), Waterlogged, River/Stream/Drain, Lakes and Ponds, Mining/Industrial areas, Reservoir/Tanks, Mangrove/Swamp Area, Crop Land, Agriculture Plantation (horticulture and agro-forestry), Core urban, Mixed settlement, Peri-urban, Village (Fig. 2).

Roads and highways were also analyzed as separate variables in the model. Roads were classified as village roads, major district roads, and state and national highways and digitized individually to estimate Euclidean distance further (Table 2). Euclidean distance from the human settlement (Core-urban, Peri-urban, villages and mixed settlement) was analyzed and taken as a separate input variable for the model.

## Supplementary Information

Supplementary Information.

## Data Availability

The datasets generated during and/or analyzed during the current study have been included in this paper and are available from the corresponding author upon reasonable request.
