# Supplementary material for: Living on the sea-coast: ranging and habitat distribution of Asiatic lions
Source: Sci Rep. 2022 Nov 10;12:19235. doi: 10.1038/s41598-022-23761-1 (PMC9649791; doi:10.1038/s41598-022-23761-1)
Supplement: Supplementary file 1 — Supplementary Information. [file 41598_2022_23761_MOESM1_ESM.docx]

**Living on the sea-coast: ranging and habitat distribution of Asiatic lions.**

**Authors: Mohan Ram*^1^, Aradhana Sahu^2^, Shyamal Tikadar^3^, Harshal Jayawant^4^, Lahar Jhala^5^, Yashpal Zala^6^, Meena Venkataraman^7^**

**1*-Corresponding author.** Deputy Conservator of Forests, Wildlife Division, Sasan-Gir, Junagadh – 362 135, Gujarat, India. [mrlegha@gmail.com](mailto:mrlegha@gmail.com) 2. Chief Conservator of Forests, Wildlife Circle, Junagadh – 362 001, Gujarat, India. [aradhanasahuifs@gmail.com](mailto:aradhanasahuifs@gmail.com) 3. Principal Chief Conservator of Forests (Wildlife) and Chief Wildlife Warden, Gujarat State, Gandhinagar – 382 010, Gujarat, India. [stikadar@gmail.com](mailto:stikadar@gmail.com)*.* 4. Geospatial analyst, Carnivore Conservation & Research (CCR), Mumbai, Maharashtra, India. [harsh.jayawant@gmail.com](mailto:harsh.jayawant@gmail.com). 5. Scientific Assistant, Wildlife Division, Sasan-Gir, Junagadh- 362 135, Gujarat, India. [laharjhala@gmail.com](mailto:laharjhala@gmail.com) 6. GIS Analyst, Wildlife Division, Sasan-Gir, Junagadh- 362 135, Gujarat, India. [yashpal1035@gmail.com](mailto:Yashpal1035@gmail.com) 7. Principal Consultant, Carnivore Conservation & Research (CCR), Mumbai, Maharashtra, India. [meena.venktraman@gmail.com](mailto:meena.venktraman@gmail.com)

**Supplementary Figure 1A and 1B**

Lion habitat distribution probability predicted for the input variable ‘Euclidean distance from road (district roads, state and national highway and village roads)’ for Lions ranging exclusively in the western coastal habitat of the Asiatic Lion Landscape, Gujarat, India, according to the MaxEnt model. The response curve is shown in different shades of black. Set of Response curves (A) show how the predicted probability of Lion presence changes as the input variable is varied, keeping all other variables at their average sample value. Set of Response curves (B) show how the predicted probability of Lion presence changes using only the corresponding variable.

**Supplementary Figure 2A and 2B**

Lion habitat distribution probability predicted for the input variable ‘Euclidean distance from road (district roads, state and national highway and village roads)’ for Lions ranging exclusively in the eastern coastal habitat of the Asiatic Lion Landscape, Gujarat, India, according to the MaxEnt model. The response curve is shown in different shades of black. Set of Response curves (A) show how the predicted probability of Lion presence changes as the input variable is varied, keeping all other variables at their average sample value. Set of Response curves (B) show how the predicted probability of Lion presence changes using only the corresponding variable.

**Supplementary Figure 1A West Response Curve A**

**Supplementary Figure 1B West Response Curve B**

**Supplementary Figure 2A East Response Curve A**

**Supplementary Figure 2B East Response Curve B**
